# Vibration Mitigation Effect Investigation of a New Slab Track Plate Design

**DOI:** 10.3390/s19010168

**Published:** 2019-01-05

**Authors:** Linya Liu, Xuan Wang, Yun-Lai Zhou, Jialiang Qin

**Affiliations:** 1 Engineering Research Center of Railway Environment Vibration and Noise Ministry of Education, East China Jiaotong University, Nanchang 330013, China; lly@ecjtu.edu.cn (L.L.); 2016018082301009@ecjtu.jx.cn (X.W.); 2015018082301012@ecjtu.jx.cn (J.Q.); 2 Department of Civil and Environmental Engineering, National University of Singapore, Singapore 117576, Singapore

**Keywords:** slab track plate, urban rail transit, optimal design, vibration mitigation, modal testing

## Abstract

This study proposed a novel vibration mitigation slab track plate design to mitigate the vibration induced in urban rail transit operations. The optimal recipe for the newly designed slab track plate is obtained by a series of laboratory tests, and both newly designed vibration mitigation slab track plates and normal slab track plates are fabricated and hereinafter tested. The newly designed slab track plate was examined with a series of laboratory tests in comparison with the normal slab track plate. The PolyMAX method is then adopted for extracting the modal properties, including resonant frequencies, and damping ratios are also determined for both the designed slab and normal slab track plates. A vibration mitigation level (*Ls*) is defined to address the vibration mitigation performance taking the acceleration response of the normal slab track plate as reference. The newly designed slab track plate demonstrated better dynamic and damping characteristics in comparison with the normal slab track plate. Under the same excitation force, the newly designed slab track plate can mitigate 8.9 dB on average in the frequency range [20, 400] Hz, expressing the feasibility of effective vibration mitigation capacity.

## 1. Introduction

Vibration is considered an essential topic in both the engineering and research communities because of its relation to structural local/global failures during lifecycle service in mechanical engineering [1], civil engineering [2,3] and aerospace engineering. With the development of urbanization, public transportation has encountered challenges such as traffic jams and demands new solutions for high traffic loads, and urban rail transit serves as an alternative for overcoming the urban transportation load. In terms of urban rail transit, its induced vibration also highly influences the daily lives of the resident close to the rail transit lines, which has also attracted the attention of the scientific community [4,5,6,7]. In [4], the effect of tunnel and soil parameters (such as tunnel depth, tunnel shape and so on) on the vibrations from underground railways was investigated to obtain the most essential parameters that contribute most to the generation and propagation of the vibrations from underground railways; in [5], a compound track model with a rail absorber is proposed by incorporating a Timoshenko beam representing the rail with a damped beam-spring system representing the absorber to address the bending mode influence. In [6], the pseudo-excitation method (PEM), symplectic mathematical scheme and Schur decomposition are adopted to resolve the moving vehicle vibration on an infinitely long periodic track. In [7], the vibrations caused by the passage of high-speed train on ballasted and non-ballasted tracks are studied to determine the ground-borne vibration mitigation performance. 

Rail transit-induced vibrations affect old architectures and any precise instruments in institutions, hospitals and universities along the rail transit lines [8]. Herein, how to decrease urban rail transit vibrations is an urgent problem to be solved at the current stage, which includes the vibration analysis from the ground and tunnel [9,10,11,12,13,14,15,16,17,18,19], rail [20], passing vehicles [21], bridges [22] and so on. In terms of ground and tunnel vibration analysis, in [9], a model for a twin tunnel embedded in a homogeneous, elastic full space is proposed to investigate the interference between tunnels in the propagation of ground-borne vibrations from an underground railway. In [10], the forced vibration of curved beams on two-parameter elastic foundation under impulsive loads is studied; for similar studies readers can refer to [11]. The ground borne vibration has been numerically analyzed for high-speed rail lines [12], and 3D coupled scaled model analysis is discussed in [13]. In [14], the forced vibration of a cracked shear deformable beam on a two-parameter elastic foundation is investigated by applying a lattice spring model. The vibration induced by railway transportation in tunnels is discussed numerically in [15]. The uncertainty quantification in the railway-induced ground vibration prediction is discussed statistically in [16]. The railway-induced ground vibration is studied by using a three-dimensional finite element (FE) model in time domain in [17]. In another study [18], a 2.5 MFS-FEM model is proposed to assess the vibrations generated by underground railway traffic. In [19], field data is utilized to anticipate the ground vibration amplitudes resulting from urban railway traffic quantitatively and qualitatively. In terms of the rails, the effects of the track irregularities on environmental vibration are investigated in [20]. The vibration of a train-bridge under track irregularities and traveling seismic waves is investigated using a train-slab-track-bridge interaction model in [21]. The train-induced vibration at high speed is analyzed in a time domain 3D model in [22,23,24]. 

Apart from using both numerical and experimental analysis for the vibration analysis in railway traffic systems as illustrated above, vibration mitigation has become an essential study focus, and a large number of investigations have been conducted for rail transit vibration mitigation [25,26,27]. In [25], the train-track-ground interaction is investigated numerically to determine the dynamic performance of ballasted tracks. Dynamic vibration absorbers are introduced to control the low frequency vibration for floating slab tracks in [26]. By combining a floating slab track and dynamic vibration absorbers, a new vibration attenuation track is developed in [27]. Li et al. [28] conducted experimental tests for the Beijing rail transit line 5, comparing the vibration mitigation performance after installation of normal fasteners, III type rail vibration mitigation fasteners, and a steel spring floating slab track. Li et al. [29] constructed a two-dimensional vehicle-rail coupling model, and compared the vibration responses for normal slab track and steel spring floating slab track under impact loading, certain moving load, and moving vehicle loading, respectively, and finally made a comparison of the vibration mitigation performances for the floating slab track under different loads. 

In [30], Wei et al. proposed an integrated approach for characterizing the dynamic behavior of wheel-rail interactions at crossings, combining the in situ axle box acceleration (ABA) and roving-accelerometer hammer tests. Ali et al. [31] proposed an image processing approach for identifying the squats automatically, which is used to model the failure risks. Both the rail defects and crack growth performance are discussed by using the data captured from video camera and ultrasonic measurements. In [32], wavelet entropy is introduced to determine local irregularities with distinct length from multiple measurements for the high-speed railway catenaries. Luis et al. [33] proposed a new prediction interval modeling technology based on fuzzy numbers and applied for load forecasting. 

In terms of all vibration mitigation analysis, the fundamental theory is the modal analysis, including experimental modal analysis (EMA), which can be dated back to decades ago [1]. The necessity of moving the testing specimens into laboratory restricts the wide application of EMA in engineering such as large scale structures such as bridges, towers and so on. The operational modal analysis (OMA) avoids such shortcoming, and paves the way for real engineering application, which only concentrate on the response analysis [2,3], where the most important is to set a reference free approach for modal properties extraction and structural characteristics determination. Transmissibility is one essential approach among all reference-free techniques [2,3], which has been widely applied in damage identification [34,35]. In [34], principal component analysis (PCA) is introduced to transmissibility for reducing the data dimension in order to improve the efficiency. In [35], the transmissibility is applied for ultrasonic testing analysis in order to remove the reference effect. 

In modal analysis, the extraction of modal properties would be of high significance and indispensable in both mechanical engineering and civil engineering, and the modal properties such as resonant frequencies and mode shapes will characterize the structures [36]. To extract the modal properties, various techniques have been proposed such as covariance-driven SSI algorithm [37], empirical mode decomposition (EMD), frequency domain decomposition (FDD), peak picking and so on [1]. The extracted modal properties shall serve for the damage identification, and vibration analysis such as vibration mitigation as well. 

Currently, the vibration mitigation for urban rail transit usually aims to optimize the fasteners and slab. The use of vibration mitigation fasteners will immediately result in rail corrugation, and simultaneously deteriorate the wheel-rail contact; on the other hand, floating slab track although it has good vibration mitigation performance, is expensive, and cannot be applied in full rail tracks. Therefore, to seek an effective but economic solution for vibration mitigation is indispensable. 

This study proposes a novel vibration mitigation slab track plate for distinct rail transit lines. The kernel for the vibration mitigation fundamentally is to utilize the internal granules from the vibration mitigation slab track plate to absorb and dissipate energy, leading to the vibration mitigation. This study designed the recipe and fabricated the new vibration mitigation slab track plate and a normal slab track plate, respectively; conducted laboratory modal testing and vibration mitigation performance tests for both slab track plate types, and compared the results hereinafter, finally discussing the vibration mitigation performance of the newly designed slab track plate.

## 2. Theoretical Background

### 2.1. Modal Analysis Using PolyMAX

In terms of a multiple-degree-of-freedom (MDOF) structural system subjecting to single load, the mathematic model can be described by the following equation:

(1) $$M\ddot{x}(t)+C\dot{x}(t)+Kx(t)=F(t)$$

where *M* means the mass, *C* indicates the damping, *K* represents the stiffness, and *F* illustrates the load, while *t* addresses the time domain. 

In modal analysis, the frequency response functions (FRFs) would be the most useful tool for extracting the modal properties such as modal frequencies, mode shapes and so on [1], expressed as:

(2) $$H_{\left(i,j\right)}(\omega )=\frac{X_{i}(\omega )}{F_{j}(\omega )}$$

where the H(ω) represent FRFs, *X* represents the dynamic responses in frequency domain, *i* and *j* represent the locations. 

EMA has single point excitation and multiple point excitations [1], while the multiple points excitation has the advantages of uniform energy distribution, low possibility in modal loss, and high accuracy, has been extensively applied for complex structures in aeronautics, ocean platforms and civil infrastructure [38]. 

The multiple point excitations PolyMAX method that has been developed in recent years can resolve the shortcomings of conventional multiple point excitations in parameter identification under strong damping and dense modes, and the results are stable and can achieve high accuracy. The analysis process can be summarized as follows:

First, we build the linear orthogonal matrix before designing the stabilization diagram, and extract the modal participator, modal frequencies and so on.

(3) $$H\left(\omega \right)=B\left(\omega \right)A{\left(\omega \right)}^{-1}$$

where A(ω) means the input matrix, B(ω) indicates the output matrix. The dimensions for H(ω) and B(ω) are both *l* × *m*, and the dimension for A(ω) is *m* × *m*:

(4) $$B\left(\omega \right)=\sum_{r=0}^{p}Z^{r}\beta_{r}$$

where $Z^{r}=e^{-j\omega \Delta t}$, Δt indicates the sampling time, *r* depicts the order. For certain frequency ω, with measured FRFs, by selecting different frequencies, then the parameters $\alpha_{r}$ and $\beta_{r}$ (*r* = 0, 1, 2, … , *p*) can be obtained as $\beta =\left[\begin{array}{l} \beta_{0}\\ \beta_{1}\\ \beta_{2}\\ \vdots \\ \beta_{p} \end{array}\right]$, $\alpha =\left[\begin{array}{l} \alpha_{0}\\ \alpha_{1}\\ \alpha_{2}\\ \vdots \\ \alpha_{p} \end{array}\right]$.

(5) $$A\left(\omega \right)=\sum_{r=0}^{p}Z^{r}\alpha_{r},Z=e^{-j\omega \Delta t}$$

where *Z* means polynomial basis functions, Δt represents the sampling interval. The parameters $\beta_{r}$, $\alpha_{r}$ can be hereinafter determined with various methods. After obtaining the above parameters, the modal contribution factor (MCF), system poles can be obtained as:

(6) $$\left[\begin{array}{ccccc} \left[0\right] & \left[I\right] & \cdots & \left[0\right] & \left[0\right]\\ \left[0\right] & \left[0\right] & \cdots & \left[0\right] & \left[0\right]\\ \vdots & \vdots & \ddots & \vdots & \vdots \\ \left[0\right] & \left[0\right] & \cdots & \left[0\right] & \left[0\right]\\ -{\left[\alpha_{0}\right]}^{T} & -{\left[\alpha_{1}\right]}^{T} & \cdots & -{\left[\alpha_{p-2}\right]}^{T} & -{\left[\alpha_{p-1}\right]}^{T} \end{array}\right]\left[V\right]=\left[V\right]\left[\overset{\wedge }{}\right]$$

By using frequency domain least square method (LSM), the mode shapes can be then obtained as:

(7) $$H\left(\omega \right)=\sum_{i=1}^{N}\left[\frac{\varphi_{i}I_{i}^{T}}{j\omega -p_{i}}+\frac{\varphi_{i}^{*}I_{i}^{H}}{j\omega -p_{i}^{*}}\right]-\frac{LR}{\omega^{2}}+UR$$

where $\varphi_{i}$ means the *i-*th mode shape, and $I_{i}^{T}$ depicts the MCF. 

### 2.2. Structural Characteristics for Slabs

In terms of the slab characteristics, especially for dynamic states, referring to the vibration mitigation level, also called insertion loss, which can address the vibration mitigation performance by using the vibration absorbers [39], a reference acceleration can be introduced to illustrate the insertion loss in rail traffic system, expressed as:

(8) $$L_{s}=20\times \log\left(\frac{a_{1\omega }}{a_{0}}\right)-20\times \log\left(\frac{a_{2\omega }}{a_{0}}\right)=20\times \log\left(\frac{a_{1\omega }}{a_{2\omega }}\right)$$

where $a_{0}$ means the reference. $a_{1\omega }$ and $a_{2\omega }$ represent the accelerations for conventional slab track plate test point and vibration mitigation slab track test point, respectively. 

In order to address the vibration mitigation effect, this study defines a novel indicator to describe it quantitatively, expressed as:

(9) $$L_{s}=20\times \log\frac{a_{1\omega }}{a_{2\omega }}$$

Apart from assessing the vibration mitigation effect, the damping ratio is also of high importance, and is determined by using a free decay test [40]. The corresponding equation is expressed as follows:

(10) ξ=ln(A1A2)2πn

where *A*_1_ and *A*_2_ represent the amplitudes of the freely decaying curves, respectively; *n* means the number of vibrations between *A*_1_ and *A*_2_. 

## 3. Design of the Novel Track Damping Plate

### 3.1. The Raw Material and the Recipe

**Figure 1:**
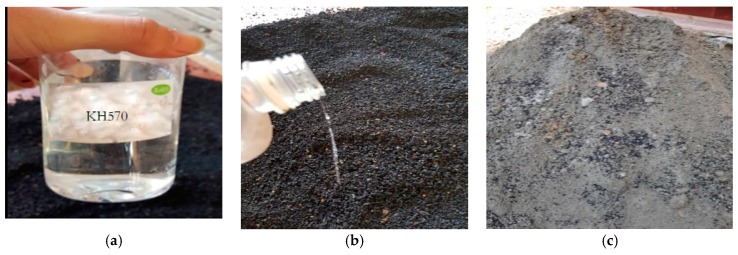
(**a**) KH570 coupling agent; (**b**) Rubber particle coupling process; (**c**) Coupled rubber particles.

In this study, ordinary Portland cement is utilized as the cementing material, for which the grading is P.O. 42.5 according to Chinese standard GB175-2007. Coupling agent and silicon rubber particles are taken to produce high damping concrete, where the coupling agent is 1.5% of rubber particles by mass. The aggregates are river sands and pebble. The mix proportion is made to Grade C45 grade concrete, where the ratio of cement paste to river sand to pebble is 1:1.65:3.84, and water to cement (*w*/*c*) ratio is 0.6. The detailed mix proportion is presented in Table 1. The term *w*/*c* means the weight ratio of used water to used cement, and *r*/*c* (rubber to cement ratio) means the weight ratio of used rubber and used cement. Figure 1 illustrates the SILANE coupling agent KH570 and the rubber particle coupling process.

### 3.2. Test Specimen Design and Mechanical Properties Testing Procedure

**Figure 2:**
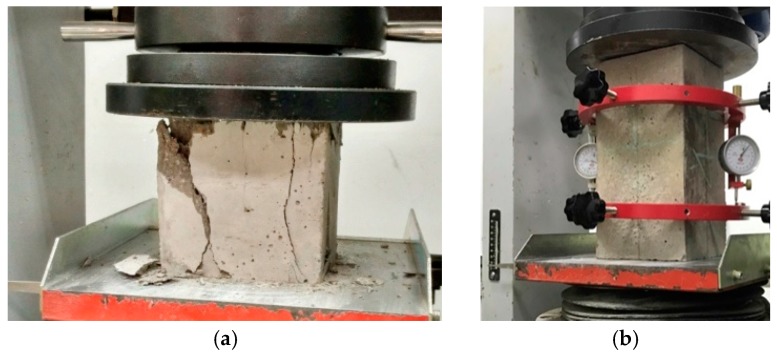
(**a**) Concrete cubic specimen compressive testing; (**b**) Young’s modulus tests for prisms specimens.

**Figure 3:**
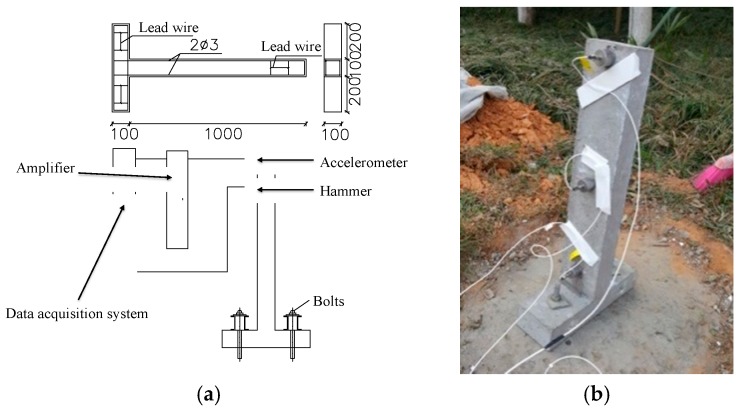
Damping ratio testing specimen and instrumentation for the rubber concrete: (**a**) Testing instrumentation diagram; (**b**) Testing instrumentation (mm).

Since this study considers the compressive strength, Young’s modulus and damping ratio for the new designed vibration mitigation slab track, three kinds of specimens were fabricated. In terms of compressive strength and Young’s modulus tests, cubic specimens with 150 mm × 150 mm × 150 mm in dimension and prisms specimens with 150 mm × 150 mm × 300 mm in dimension are fabricated referring to the code [41] shown in Figure 2. In terms of the concrete damping ratio tests, currently no codes can be found. This study follows the previous investigation [42,43]. *T* shape cantilever specimens are taken as depicted in Figure 3, the test length is 1000 mm, the section area is 100 mm × 100 mm, the installation part has 500 mm in length; the section area is 100 mm × 100 mm.

During testing, anchor bolts are utilized to fasten the cantilever beam to the base trough; hammer excitation was applied at the cantilever beam. The vibration shall fade because of damping. The vibration responses were captured by BK-4507 accelerometers as shown in Figure 3b and were transferred by an ADBZ2101amplifier to a LMS310 data acquisition system. Fast Fourier Transform (FFT) was applied to obtain amplitude spectra in the frequency domain, and a single frequency curve in the frequency domain is determined after filtering. The above damping equation (Equation (10)) will yield the damping ratio. The test specimens and instrumentation are described in Figure 1. In order to avoid concrete fractures during the tests, symmetrical reinforcing bar with 2Ø3 steel bar was applied, and hoop reinforcement adopted No. 8 lead wire; the space distance is 50 mm.

### 3.3. Mechanical Properties Analysis

Tests for the tensile strength and Young’s modulus were conducted in laboratory, and results are summarized in Table 2. For the purpose of comparing the concrete performance between different recipes, the damping ratio is also measured and illustrated in Table 2. 

From Table 2, increasing the rubber to cement (*r*/*c*) ratio could increase the concrete damping ratio significantly and reduce the Young’s modulus. However, the compressive strength is also impaired at the same time. At the same *r*/*c* ratio, adding coupling agent could improve the concrete strength to some extent. In order to obtain both concrete strength and damping property, it is indispensable to add coupling agent to the mix and control the *r*/*c* ratio. The testing results demonstrated that, after adding coupling agent, when the *r*/*c* ratio increased from 0% to 12%, the concrete damping ratio increased from 25.4% to 120.4%, and the Young’s modulus was reduced from 12.5% to 27.0%, but the concrete strength decreased 3.7% to 37.4%. When the *r*/*c* ratio exceeded 6%, concrete strength rapidly dropped from 9.3% to 21.1%. Hence, in overall consideration of concrete strength and damping property, the optimal mix proportion was selected as follows: *w*/*c* was 0.6, *r*/*c* was 6%, coupling agent was 1.5% in mass, and cement paste to river sand to pebble was 1:1.65:3.84. In this study, the above optimal mix proportion was adopted to cast the high damping concrete and produce the novel vibration mitigation slab track plate design. 

## 4. Modal Analysis for the Novel Track Damping Plate

### 4.1. The New Designed Vibration Mitigation Slab Track Plate Casting

This study designed a new vibration mitigation slab track plate by casting the rail supporters and rail slab into one, and this can apply for different rail situations such as bridges, roadbeds, and tunnels. 

**Figure 4:**
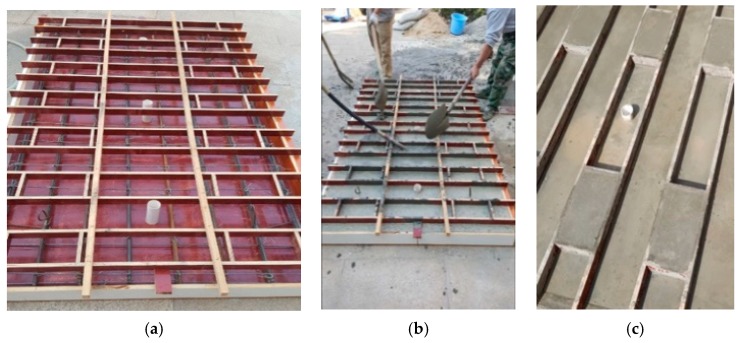
(**a**) Reinforcing bar setting; (**b**) Casting processing; (**c**) Casted slab track plate.

**Figure 5:**
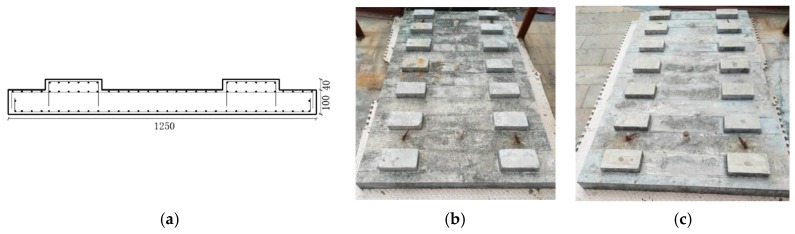
(**a**) Reinforcing bar diagram; (**b**) Newly designed slab track plate; (**c**) Normal slab track plate (mm).

In order to compare the free mode and vibration mitigation performance between the new designed slab track plate and the normal slab track plate, the length, width and height were modified to be half of a real engineering rail slab track plate. The concrete recipe #1 in Table 1 was considered to fabricate normal slab track plate, and the optimal designed recipe discussed in Section 3.1 was also considered for fabricating a newly designed slab track plate. Figure 4 described the slab track plate casting process. Figure 5 illustrates the reinforcing bar diagram for the slab track, and fabricated specimens of newly designed slab track and normal slab track, respectively. For both specimens, the dimensions were 2230 mm × 1250 mm × 10 mm.

### 4.2. Test Instrumentation

**Figure 6:**
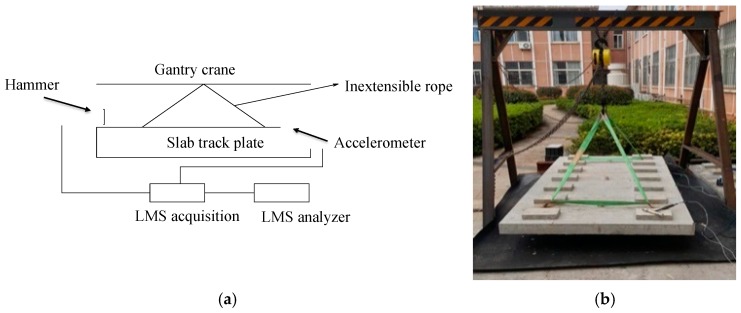
(**a**) Testing diagram for slab track plate; (**b**) Free-free mode testing instrumentation.

The modal testing system for the slab track consists of three parts: excitation, data acquisition system, and parameter identification system as shown in Figure 6. The excitation source uses a PCB 086D05 hammer, with resolution 0.23 mv/N, ranges ±22 kN pk. The data acquisition system utilizes a Belgian LMS310 data acquisition system (24 channel data acquisition system, A/D24), and the sensors are considered as accelerators. The parameter identification system utilizes the PolyMAX method from the Impact Testing module in the LMS system.

In order to guarantee the absence of constraints, this study adopted an inflexible rope for hanging the slab plate specimen from a gantry crane, and keep the slab plate horizontally stable. Because the inflexible rope is lightweight, which should not influence the slab plate mode, then the slab plate could be considered as being under constraint-free conditions. 

### 4.3. Modal Testing Setup

**Figure 7:**
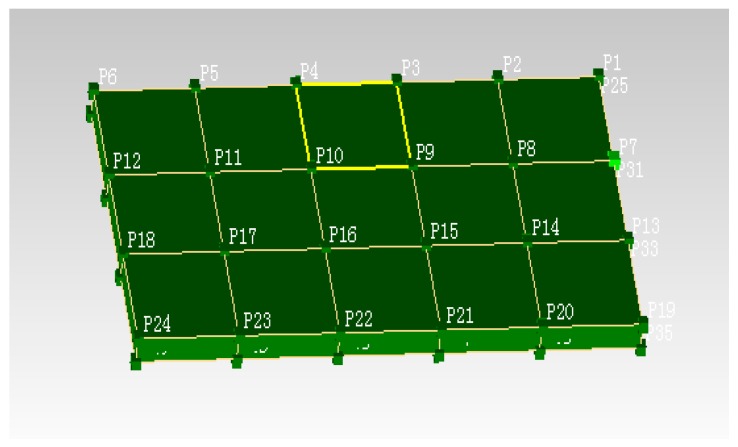
Testing point distribution for the slab plate.

The rail plate is initially analyzed to extract the structural dynamic characteristics. The testing point is then chosen for the peak of the mode shapes using modal testing theories [44], and it should be avoided to select the stationary point of the mode shapes. This test utilized averaged spaced testing points with six testing horizontal points and four vertical testing points, as depicted in Figure 7.

Due to the limiting use of sensors, this study applied the single excitation, multiple points’ measurement modal separation method [45], and fully adopted excitation and measuring point’s selection to separate the modes, finally this study made the complex structure behave as simple mode. By acting a vertical excitation at P8, measuring the vertical responses of other points will extract the vertical vibration mode; by acting a horizontal excitation at P18, measuring horizontal responses of other points will extract the lateral vibration mode. 

### 4.4. Modal Analysis

This section applied the PolyMAX method to obtain the modal parameters for the tested slab track plates discussed above, and Table 3 shows the first six order modal parameters in comparison with the analytical results. From Table 3, the errors between the testing results and analytical results are very small, and the mode shapes from the tests coincide with the analytical results. This implies that the hammer excitation to the slab plate has good accuracy in modal testing. 

**Figure 8:**
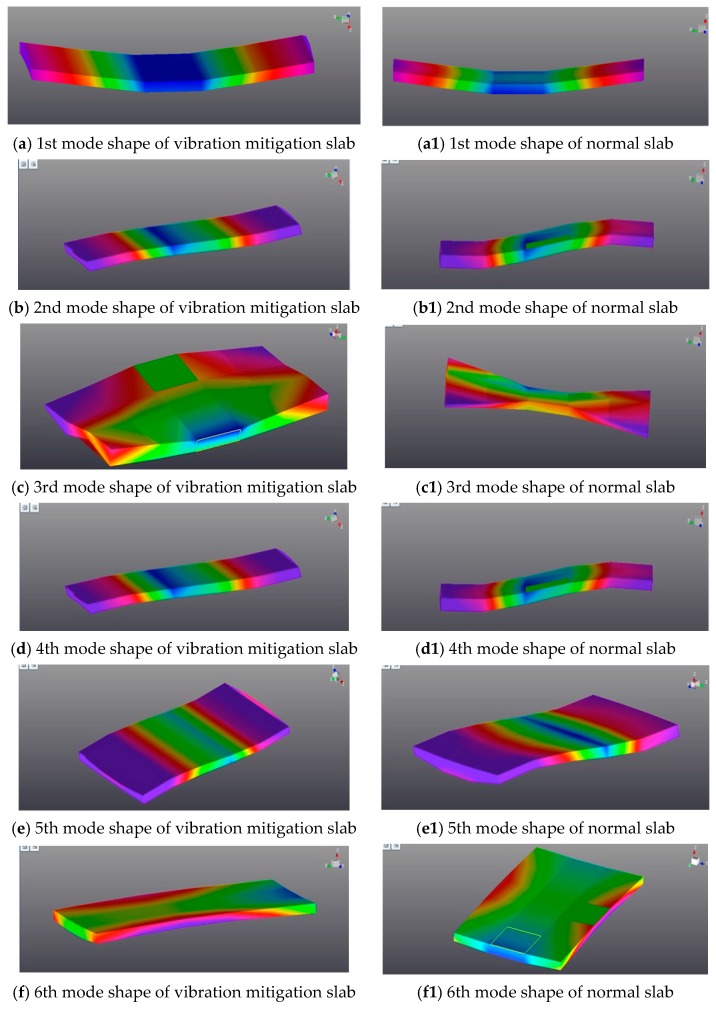
The comparison between rubber vibration mitigation slab and normal concrete slab.

From the modal testing results analysis, under the same structural style and same constraint condition, the first six mode shapes of the vibration mitigation slab track plate agreed well with those of the normal slab track plate as shown in Figure 8. However, the resonant frequencies of vibration mitigation slab track plate all are smaller than the corresponding resonant frequencies of normal slab track plate from Table 3, and the damping ratios for vibration mitigation slab plate are all bigger than those for normal slab plate, which implied that both the dynamic characteristics and damping characteristics of the vibration mitigation slab track plate have been greatly improved. This means that the newly designed slab track plate has better energy dissipation performance, thus leading to the better vibration mitigation.

## 5. Vibration Mitigation Analysis for the New Designed Track Damping Plate

### 5.1. Testing Setup

**Figure 9:**
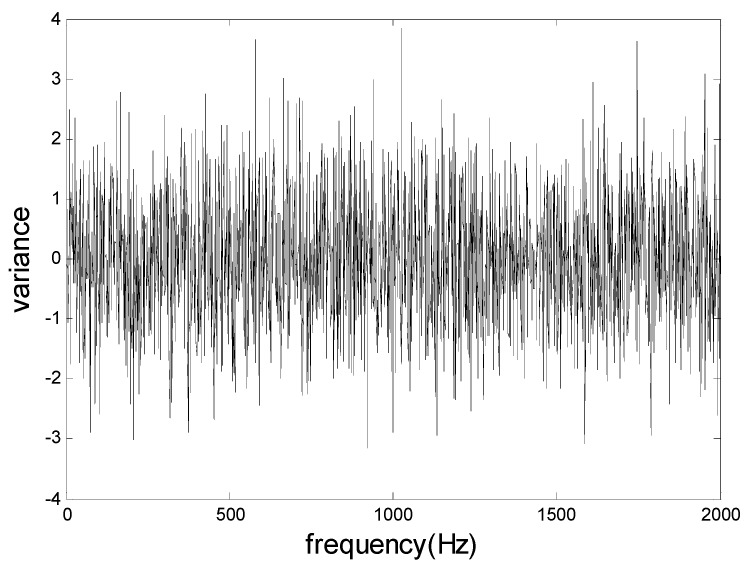
The white noise excitation in frequency domain.

The new designed vibration mitigation slab plate test adopted a Head Recorder data acquisition system, the HEV-200 exciter applied an excitation force of 200 N, and the amplitude was ±10 mm, the sensors are PCB356A16 three directional accelerometers with 100 mv/g resolution; the range is ±50 g with frequency band [0.5, 5k] Hz. This study tried to compare the vibration responses between the new vibration mitigation slab plate and a normal slab plate. In order to keep the same excitation, the vibration mitigation performance tests applied the same excitation force at the same location for the vibration mitigation slab plate and normal slab plate, respectively. Excitations include transient excitation, random excitation, swept sine excitation and so on [46]. This study adopted the white noise from the random excitation as excitation force shown in Figure 9. The power at each frequency band for the white noise is the same, and the energies acting at both slabs are the same. 

### 5.2. Tests and Results Analysis

**Figure 10:**
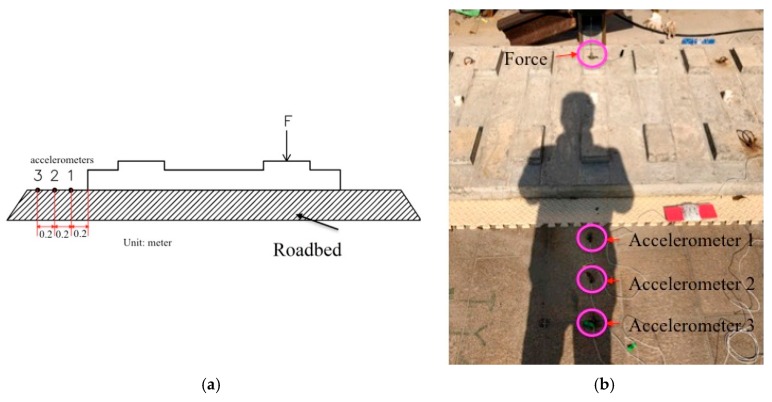
(**a**) The diagram for the testing points distribution (unit: m); (**b**) Testing instrumentation.

By fixing the two slabs on a roadbed, and using an excitation machine we conducted the loading tests for the slabs. Accelerometers are placed at measuring points 1, 2 and 3. Testing points 1, 2, and 3 are located 0.2 m, 0.4 m, and 0.6 m at the side of slab, as shown in Figure 10a. Testing process and measuring points are exactly the same for the two slabs as shown in Figure 10b. 

**Figure 11:**
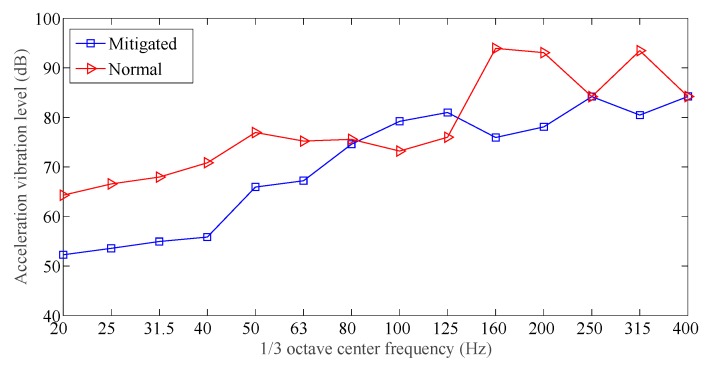
Acceleration spectrum for testing point 1.

**Figure 12:**
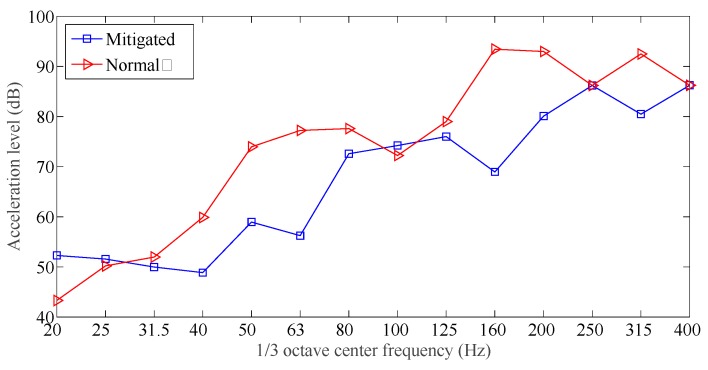
Acceleration spectrum for testing point 2.

**Figure 13:**
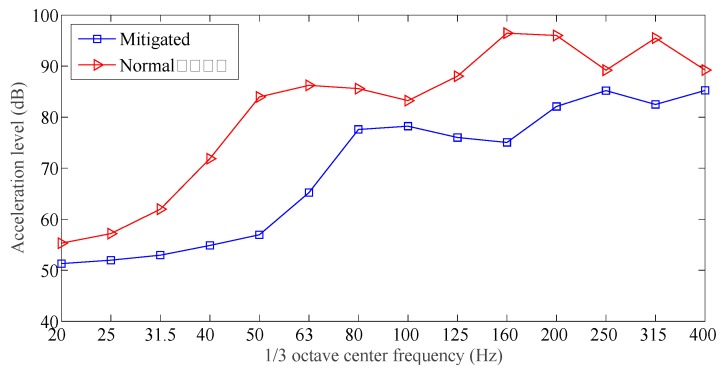
Acceleration spectrum for testing point 3.

The time domain responses for all the measuring points 1, 2, and 3 are obtained during the tests, acceleration spectrum curves were obtained by applying the FFT to the measured responses. Figure 11, Figure 12 and Figure 13 showed the acceleration spectrum curves, and Table 4 summarizes the acceleration vibration level.

From Figure 11, Figure 12 and Figure 13 and Table 4, when an excitation force is applied to both slab track plates, the maximum vibration level and averaged vibration level at the measuring points were all much smaller than those at the normal slab track plate. In the frequency range [20, 400] Hz, except for 20 Hz and 100 Hz, the vibration mitigation slab track plate did not show a clear vibration mitigation capacity; but for all other frequency bands, the vibration levels achieved [5, 25] dB. In all frequency bands, the vibration mitigation slab track plate obtained an averaged vibration mitigation of 8.9 dB, which implied that the new designed vibration mitigation slab track plate has good vibration mitigation capacity. 

**Figure 14:**
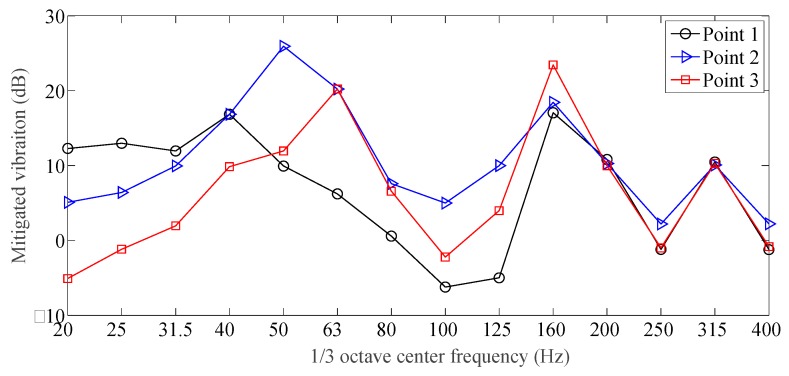
Mitigated vibration for the vibration mitigation slab track plate.

From Figure 14, the new designed vibration mitigation slab track plate has different vibration mitigation performance at different frequency bands: in the frequency range [20, 80] Hz and above 100 Hz, the vibration mitigation capacity is outstanding; in the frequency range [80, 100] Hz, the vibration mitigation slab track plate slightly amplified the roadbed vibration. The main reason is that the new designed slab track plate included damping granules, and produce a certain resonant frequency of the slab track plate-road foundation system within such a frequency range, leading to the slight vibration enlargement. From Figure 14, the overall vibration mitigation curve located in the positive range, the minimum is −6.5 dB, and the maximum is 26.8 dB. The averaged vibration mitigation for the vibration mitigation slab track plate is 8.9 dB, which implied that the vibration mitigation slab track plate performs well in vibration mitigation. 

## 6. Conclusions

This study discussed the optimal design for a new vibration damped slab track plate based on test verification, and obtained a new recipe for the slab track plate design, and hereinafter fabricated the new designed vibration damped slab track plate and a conventional normal slab track plate, respectively. Free-free modal testing and vibration mitigation capacity tests were conducted and compared. The conclusions can be summarized as follows:

(1) The introduction of silicon rubber could promote the damping ratio of the new vibration mitigation slab track plate but reduces its strength, but the coupling agent can compensate the strength at certain amount. For the purpose of balancing the mechanical properties and damping characteristic, it is indispensable to keep the *r*/*c* to be 6%, and adding 1.5% by of coupling agent.
(2) Under the same constraint conditions and structural style, the mode shapes of the newly designed vibration mitigation slab track plate overall agreed with those of the normal slab track plates, but each resonant frequency for the vibration mitigation slab track plate is less than that for the normal slab track plate; each modal damping for the vibration mitigation slab track plate is higher than that for the normal slab track plate. This means that the dynamic characteristics and damping properties are all enhanced.
(3) In laboratory testing, under excitation loading, the new vibration mitigation slab track plate has better vibration mitigation performance, with a maximum vibration mitigation of 26.8 dB, and an average vibration mitigation of 8.9 dB, which implies the newly designed slab track plate has better vibration mitigation capacity. The vibration mitigation performance for this newly designed slab track plate under real rail transit lines needs further investigation.

## Figures and Tables

**Table 1:** The recipe for high damping concrete.

Concrete Recipe	*w*/*c* Ratio	*r*/*c* Ratio/%	Cement Weight (kg)	Silicon Rubber Weight (kg)	Coupling Agent/%
#1	0.6	-	308	0	-
#2	0.6	3	308	10	-
#3	0.6	3	308	10	1.5
#4	0.6	6	308	20	-
#5	0.6	6	308	20	1.5
#6	0.6	9	308	40	-
#7	0.6	9	308	40	1.5
#8	0.6	12	308	80	-
#9	0.6	12	308	80	1.5

**Table 2:** Test results for high damping concretes.

Concrete Specimen	Tensile Strength/MPa	Young’s Modulus/GPa	Damping Ratio/%
#1	46.5	35.9	2.05
#2	43.5	31.9	2.55
#3	44.8	31.4	2.57
#4	38.4	30.2	3.31
#5	42.2	29.8	3.44
#6	31.2	27.5	3.92
#7	36.7	27.9	4.06
#8	23.3	26.4	4.39
#9	29.1	26.2	4.52

**Table 3:** Modal frequencies and damping ratio comparison.

Order	Vibration Mitigation Slab Plate				Normal Slab Plate			
	Resonant Frequency/Hz			Damping Ratio/%	Resonant Frequency/Hz			Damping Ratio/%
	Test	FEA	Error/%		Test	FEA	Error/%	
1	65.771	64.940	1.19	0.89	85.084	84.530	0.66	0.42
2	75.211	73.290	2.62	0.87	97.297	96.020	1.33	0.48
3	161.115	160.250	0.54	0.98	208.435	204.83	1.76	0.45
4	179.393	175.680	2.11	0.91	231.004	229.79	0.53	0.47
5	201.462	199.450	1.01	0.85	266.271	268.360	0.78	0.49
6	248.508	250.420	0.76	1.04	327.284	330.910	1.10	0.56

**Table 4:** The acceleration level for testing points.

Measuring Points	Designed Damping Slab (dB)		Normal Track Slab (dB)		Average Difference
	Maximum	Average	Maximum	Average	
1	85	70.5	93	77.9	7.4
2	86	66.5	92	73.5	7.0
3	84	58.6	96	70.8	12.2
